# Common conditions of use elements. Atomic concepts for consistent and effective information governance

**DOI:** 10.1038/s41597-024-03279-z

**Published:** 2024-05-08

**Authors:** Maria del Carmen Sanchez Gonzalez, Pim Kamerling, Mariapia Iermito, Sara Casati, Umar Riaz, Colin D. Veal, Monika Maini, Francis Jeanson, Oussama Mohammed Benhamed, Esther van Enckevort, Annalisa Landi, Yanis Mimouni, Clèmence Le Cornec, Domenico A. Coviello, Tiziana Franchin, Francesca Fusco, Jose Antonio Ramírez García, Loes F. M. van der Zanden, Alexander Bernier, Mark D. Wilkinson, Heimo Mueller, Spencer J. Gibson, Anthony J. Brookes

**Affiliations:** 1 Institute for Rare Diseases Research (IIER), Instituto de Salud Carlos III, Av. Monforte de Lemos, 5 PC, 28029 Madrid, Spain; 2 VASCERN ERN & Radboud University Medical Center - Center for Radiology and Nuclear Medicine, Geert Grooteplein Zuid 26-28 (route 260), 6525 GA Nijmegen, The Netherlands; 3 Istituto Neurologico “Carlo Besta” |, Fondazione IRCCS Via Giovanni Celoria, 11, 20133 Milano, MI Italy; 4 BBMRI-ERIC, Neue Stiftingtalstrasse 2/B/6, 8010 Graz, Austria; 5 Dept. Genetics and Genome Biology, University of Leicester, University Road, Leicester, LE1 7RH UK; 6 Ontario Brain Institute, Centre for Analytics, 1 Richmond St. West, Suite 400, Toronto, Ontario M5H3W4 Canada; 7 Departamento de Biotecnología-Biología Vegetal, ETSI Agronómica, Alimentaria y de Biosistemas, Centro de Biotecnología y Genómica de Plantas (CBGP, UPM-INIA/CSIC), Universidad Politécnica de Madrid, Madrid, 28040 Spain; 8 Department of Genetics, University Medical Center Groningen, University of Groningen, Hanzeplein 1, 9713GZ Groningen, Nederland; 9 Fondazione per la Ricerca Farmacologica Gianni Benzi onlus, Via Giulio Petroni, 91/D, 70124 Bari, Italy; 10 Thematic Institute of Genetics, Genomics & Bioinformatics, INSERM, Paris, 75013 France; 11 Pediatric Nephrology Division, Center for Pediatrics and Adolescent Medicine, Heidelberg University Hospita, Im Neuenheimer Feld 130.3, 69120 Heidelberg, Germany; 12 Dept. Laboratory of Human Genetics, IRCCS Istituto Giannina Gaslini, Via Gerolamo Gaslini 5, 16147 Genova, Italy; 13 Research Biobank, IRCCS Bambino Gesù Children’s Hospital, Rome, Italy; 14 Institute of Genetics and Biophysics “Adriano Buzzati- Traverso” IGB-ABT, Department of Biomedical Sciences (DSB), National Research Council (CNR), Via P. Castellino 111, 80131 Naples, Italy; 15 ERN for Rare Urogenital Diseases and Complex Conditions (ERN eUROGEN), department of Urology & IQ Health science department, Radboud university medical center, Geert Grooteplein Zuid 10, 6525 GA Nijmegen, the Netherlands; 16 Centre of Genomics and Policy, McGill University, Faculty of Medicine and Health Sciences, 740, avenue Dr. Penfield, suite 5200, Montreal, Quebec H3A 0G1 Canada

**Keywords:** Standards, Software

## Abstract

Myriad policy, ethical and legal considerations underpin the sharing of biological resources, implying the need for standardised and yet flexible ways to digitally represent diverse ‘use conditions’. We report a core lexicon of terms that are atomic, non-directional ‘concepts of use’, called Common Conditions of use Elements. This work engaged biobanks and registries relevant to the European Joint Programme for Rare Diseases and aimed to produce a lexicon that would have generalised utility. Seventy-six concepts were initially identified from diverse real-world settings, and via iterative rounds of deliberation and user-testing these were optimised and condensed down to 20 items. To validate utility, support software and training information was provided to biobanks and registries who were asked to create Sharing Policy Profiles. This succeeded and involved adding standardised directionality and scope annotations to the employed terms. The addition of free-text parameters was also explored. The approach is now being adopted by several real-world projects, enabling this standard to evolve progressively into a universal basis for representing and managing conditions of use.

## Introduction

There is a widespread desire to maximise the use and re-use of biomedical data and samples. This ambition is, however, plagued by many complex and diverse issues pertaining to information governance. To be conducted in a responsible manner, the sharing and access of data and samples must take account of many constraints, not least individual consent, requirements set by custodians (researchers, institutions) and funders, policies set by ethics committees, and legal considerations. Beyond straightforward cataloguing of shareable datasets in archives, such as the National Centre for Biotechnology Information’s database of Genotypes and Phenotypes (dbGaP) and the European Genome-phenome Archive (EGA), the wider and more challenging world of data governance operates on an inconsistent and poorly structured basis.

Numerous general policy documents and guidelines have been created in recent years, which do a great job of establishing general principles for data and sample sharing. But these resources typically fail to provide a sufficiently fine-grained and concrete basis for operationalising the recommended principles. Custodian organisations currently have not universally adopted a standardised or consistent way to structure or express the wide array of information that might be part of their sharing and access policies. Consequently, the field is challenged by considerable diversity regarding consent form design and content, data sharing agreement clauses, choices over licensing models, institutional data sharing rules, and data management plans. This is not to say that specific elements of such tools should always or often be identical, as there will always be a need for context-specific variation on such matters. Nevertheless, more consistency and standardisation are needed at the level of specific concept structuring. That is, below the overarching general policy level, and above the detailed clause and textual level, there needs to be more uniformity and agreement over what would be a useful (non-redundant, unambiguous, sufficiently comprehensive) list of governance considerations and parameters.

Creating a fully comprehensive and universally applicable ‘conditions of use’ concept list for the whole biomedical domain would be unrealistic. But to make progress one could break down this semantic challenge to address distinct governance use cases, and then concentrate on the most widely used/needed concepts and parameters. This would bring substantial and rapid impact. The most significant previous efforts in this direction would be the Consent Codes list of permitted and conditional forms of data use^1^, and the further development and improvement of this as represented by the Data Use Ontology^2^ (DUO). These approaches focus primarily on capturing headline conditions for acceptable sharing of genomics datasets and reflect the concepts and combinations of concepts that emerged organically from the field. DUO and similar ontologies are intended to capture common permissions inherent in biomedical research data. Ontologies of this nature are especially useful where research consortia prospectively design informed consent materials and data governance strategies to be compatible with selected ontology terms. However, there remains a demonstrated need for flexible systems that can capture complex and conditional permissions in data, in a manner that enables logical computer-based reasoning. This class of systems may be necessary to represent and to compare permissions inherent in numerous categories of datasets, such as legacy datasets for which data governance rules have already been generated, or those that are subject to regulatory requirements that can only be described using contingent and conditional language.

Another related initiative is the “Automatable Discovery and Access Matrix” (ADA-M)^3^. This approach emphasised the design of a data structure into which conditions of use information could be placed. To that end it included a list of concepts of use, but these were largely based on the Consent Codes and DUO terms, and these were not rigorously validated by community testing. In addition, much of the substantive information conveyed in an ADA-M profile regarding permissible and impermissible data uses is articulated through its free text fields. Consequently, ADA-M cannot enable the machine-actionable discovery of data according to its data governance conditions.

Given the above, and with the European Joint Programme for Rare Diseases (EJP-RD) stated aim: “…. to create an effective rare diseases research ecosystem for progress, innovation and for the benefit of everyone with a rare disease. We support rare diseases stakeholders by funding research, bringing together data resources & tools, providing dedicated training courses, and translating high quality research into effective treatments” (https://www.ejprarediseases.org/), we worked to devise a rational lexicon for “conditions of use” information. Our aim was to meet the need within the programme to convey the use conditions associated with the various bioresources, in a convenient and automatable way. Further, it should be easy to deploy requiring only minimal training, with profiles that are human readable as well as being able to be represented in a computer readable format. To this end the resultant system needed to be able to encode use conditions that were applicable to data and biosamples.

The resulting design is called “Common Conditions of use Elements (CCE)”, representing a highly validated set of atomic (non-overlapping) concepts of use that offer a robust and consistent basis to many data governance tools and activities. CCE is explicitly not seeking to be an ontology (though it could inform future extensions to existing ontologies), but to be a categorisation framework for conditions of use concepts. As a version 1.0 product, the hope is that various groups in different settings will adopt and provide feedback on its utility, enabling it to be progressively improved.

## Results

To produce a useful CCE list, efforts started on the domain of rare disease related registries, biobanks, and data repositories - especially as related to European Reference Networks (ERNs)^4^.

We identified four principles that are necessary for the construction of the CCE concepts:

1. be atomic, i.e., represent an operationally pure and singular concept, (*elemental*).
2. have no directionality, i.e., not convey any indication about whether that mode of use is allowed, forbidden, or obligatory, (*neutrality*).
3. be generalised, i.e., be a modular category of use that states no customisation details, conditionalities or dependencies, (*generic*).
4. be widely applicable and relevant, (*relevance*).

By way of example, consider a potential CCE category such as “Regulatory Jurisdiction”. This meets design principle #1 because it is not conflated with any other concept, such as “Geographical Area”. It meets design principle #2 because it does not attempt to covey whether data/sample use is permitted or forbidden in certain Regulatory Jurisdictions. It meets design principle #3 because it does not name any specific Regulatory Jurisdictions or imply the presence or absence of any modifying considerations (e.g., the degree of anonymity of the data). And it meets design principle #4 because it is a widely relevant consideration. Hence, it is simply a pure, generalised, categorisation concept pertaining to conditions of use of some un-named artifact.

The initial set of suggestions for CCE list items contained 76 preliminary concepts, produced after the independent extraction of all possible concepts from a series of domain consent forms, and material/Data transfer agreements (MTAs/DTAs) and after the elimination of duplicates. Each of the 76 concepts was characterised as being primarily a ‘criterion’ or ‘boundary conditions’ of use (‘who’, ‘what’, ‘why’, ‘when’, ‘where’ – 46 such elements), versus mainly a ‘process’ or ‘method’ of use (‘how’ – 19 such elements), or others not fitting into either group and so not further evaluated as a possible CCE item (11 such elements). After this categorisation, we debated and refined the list, according to the four principles above.

An overview of the analysis process, with the initial set of CCE terms can be found at the supplementary Table 1, that also summarises the process of refinement and criteria.

Directional items were made non-directional. For example, the Opt out form for Genomics England has the concept of “Patient has requested NOT to receive additional findings” in part 7B,was deemed to have the core concept of “return of incidental findings”. This concept can then be forbidden using an appropriate rule. Similarly, to meet the requirement of being atomic non-atomic items were split into their component parts. As an example, one of the ERN registries forms, (that relate to how the resources can be used) has the use condition of “recontacting the participant to return results or incidental findings” within the conditions that a participant must agree to. This term was split into the 3 CCE terms: (Re-)Identification, Return Of Results and Return Of Incidental Findings.

To make the resulting system more broadly applicable, the group also considered that some of the resources may condition use upon (i) the commercial or non-commercial character of the entities that use the data, and (ii) whether or not the intended use is profit-motivated. These concepts are typically conflated and or one is omitted (with conflation being implied/inferred) in alternative approaches.

The resulting 14 core concepts from this review and refinement process, were given a definition and a priority ranking based on anticipated utility and degree of relevance to the targeted use case (i.e., factors relevant to the secondary user of data or samples).

Based on community suggestions we added in 4 extra concepts that had not been surfaced by the starting set of consent forms and MTAs. Two of the original CCE terms were interpreted in different ways by the testers (Clinical Use and (Re-)Identification) indicating that they were not truly atomic in nature. Hence, these terms were split as discussed below, which resulted in 6 new terms in total.

By rounds of alpha-testing and consultation with the intended user community we progressively distilled the list down to what was felt to be a practically useful and usable size. The final version 1.0 CCE list comprises 20 items, as presented in Table 1 (criterion type terms) and Table 2 (process type terms). The modifications to the initial list of CCE terms are stated in the subsequent paragraph.

**Table 1:** Full list of criterion type CCE terms.

Concept	Definition
Clinical Care Use^b^	Use for patient healthcare and related services.
Clinical Research Use^b^	Use for research-related activities that involve human subjects where the intention is to advance medical knowledge.
Commercial Entity^a^	Use by an entity in the commercial sector, whether or not that use seeks to make a financial profit.
Disease Specific Use^a^	Use for research-related activities pertaining to one or more specific diseases or disease categories.
Geographical Area^a^	Use within specified geographic region(s)
Profit Motivated Use^a^	Use with the intention of making profit.
Regulatory Jurisdiction^a^	Use within an area defined by a shared legal framework, or subject to a common oversight organisation.
Research Use^a^	Use for research-related exploration or innovation.
Use As Control^a^	Use as a reference, benchmark or normal control for research or other activities.

^a^Original concept (first iterative rounds); ^b^originated by decomposition of one original concept (further iterative rounds).

**Table 2:** Full list of process type CCE Terms.

Concept	Definition
Collaboration^a^	Use that involves some form of collaboration, typically with the resource provider.
Ethics Approval^c^	Use involves a requirement on the recipient to evidence suitable ethics board (e.g., IRB/ERB) or other intuitional or oversight body approval.
Fees^a^	Use that involves payment as a basis for access or use.
Publication Moratorium^c^	Use involves a requirement on the recipient to not publish derived results before a specific date, time period, or other condition (such as approval from the supplying institution) has been met.
Publication^c^	Use involves a requirement on the recipient to make derived results available to the wider scientific community.
(Re-)Identification Of Individuals Mediated By The Resource Provider^b^	Use of records or samples in a resource (provided in a non-identified form) in a manner that identifies or re-identifies one or more individuals, mediated with the involvement of the resource provider.
(Re-)Identification Of Individuals Without Involvement Of The Resource Provider^a^	Use of records or samples in a resource (provided in a non-identified form) in a manner that identifies or re-identifies one or more individuals, without the involvement of the resource provider.
Return Of Incidental Findings^a^	Use that involves a requirement on the recipient to return results that were not intentionally generated by the planned use, to the resource provider.
Return Of Results^a^	Use that involves a requirement on the recipient to return results that were intentionally generated by the planned use, to the resource provider.
Time Period^a^	Use that has some time-frame limitation.
User Authentication^c^	Use involves a requirement on the recipient to successfully undertake some form of ID proofing and authentication, prior to the access or use.

^a^Original concept (first iterative rounds); ^b^originated by decomposition of one original concept (further iterative rounds); ^c^Added by the community users (last iterative round and validation rounds).

Some considerations that went into producing the full list of CCEs in consultation with the alpha test group, were as follows:

### CCE Terms: Geographical area and regulatory jurisdiction

Initially only the term geographical area was proposed, as it was thought that this could encompass both concepts, however with jurisdictions such as the EU spanning complex geographies, we concluded that to maintain the atomic nature of CCE these two concepts needed to be separated.

### CCE Terms: Clinical care use and clinical research use

The initially suggested term of “Clinical Use” was highlighted by the alpha testers as being too vague, in that it covered both clinical research use AND clinical care of the patient. This led to the splitting of the term into “Clinical Research Use” and “Clinical Care Use”.

### CCE Terms: (Re-)Identification of individuals without the involvement of the resource provider and (Re-)identification of individuals mediated by the resource provider

A single (Re-)Identification term was initially introduced to cover any activity where a resource was used in a manner that allowed the reversing of anonymisation/pseudonymisation. It was assumed that this would always be forbidden, and “safeguards” would apply during use to prevent this occurring. However, we found that alpha testers interpreted this term in two distinctly diverse ways. One was to forbid the re-identification of participants as expected, while the other related to ways that the participant could be re-contacted (via the supplying institution) for some legitimate purpose such as recruitment to further studies. Hence the two CCE terms were devised to capture these two separate concepts of use.

### Terms added based on feedback from alpha testers

The following CCE terms were added to cover the full range of concepts deemed important by alpha testers: Publication Moratorium, Publication, User Authentication, and Ethics Approval.

### Validation of the final 20 cce terms by creation of regularised sharing/access policies

As a final validation exercise for CCEs against our intended use case, four biobanks, three patient registries and a data platform were asked to try to use the CCE lexicon as a basis for structuring an overview (called a Policy Profile) of their main sharing policy items. Guidance was prepared to support this task, in which it was made very clear that the goal was:

- To base these Policy Profiles upon the 20 CCE concepts, each of which could be used zero or more times according to what they wanted to express in their policy.
- To represent only those Policy Profile items applicable to the secondary user of the data or samples, not those that apply to the custodian biobank/registry themselves.
- To accomplish the exercise by merely adding a directionality statement (“Permitted”, “Obligated”, “Forbidden” or ‘‘No Requirements’’) and a “scope” indicator (“Whole of resource” or “Part of resource”) to the employed CCE items. This approach leverages the Digital Use Conditions (DUC)^5^ Schema for expressing conditions of use information which was recently developed by an IRDiRC Task Force. The DUC schema employs the term “asset” to refer to both a collection of items or a singular item, being made available for some form of use. In contrast, since the CCEs in this current work are designed to support the creation of Policy Profiles for whole organisational structures (e.g., biobanks or RD registries) we instead use the term “resource”.

Supplementary Table 2 contains the feedback from validators on all CCE Terms. Overall, the process was very straightforward, with two major take-home lessons emerging. First, organisations can have very different objectives in mind when creating such Policies Profiles – ranging from wanting to be quite comprehensive in stating what can and cannot be done with their data or samples, through to others that merely wanted to provide a minimal list of headline “showstopper” categories of use that could never be allowed. Second, most groups were rather cautious about any public release or exposure of their Policy Profiles (we even had to adapt our support tools because of this concern, so that no profile was ever left on a public server) - the implications of which probably merit further investigation. Nevertheless, despite the diversity of objectives, CCEs were able to accommodate all these approaches. Additionally, there was no request for any additional CCE concepts to be added.

Policy Profiles such as these demonstrate how well CCEs can provide the basis for a consistently clear and structured representation of access policies across many organisations. However, they may not provide sufficient details for some more-demanding applications (e.g., Data Access Committee deliberations). Therefore, as one last final test of CCEs, the alpha-tester organisations kindly generated some additional free-text parameters to supplement and elaborate a number of the term + rule + scope triads previously provided in their Policy Profiles. These have been collated into a set, that gives an example for each CCE term from the free texts (Table 3 for criterion type terms and Table 4 for Process type terms) where provided.

**Table 3:** Criterion type CCE terms with further elaboration.

CCE Term	Rule	Scope
Further parameters		
**Commercial Entity**	**Permitted**	**Part of Resource**
Permitted only if it has been authorised in the informed consent.		
**Clinical Care Use**	**Permitted**	**Part of Resource**
Use for diagnosis purpose, only if a specific authorisation in the informed consent is present.		
**Clinical Research Use**	**Permitted**	**Part of Resource**
Use for research purposes, including research to improve diagnosis and treatment, in the field of the disease for which the biological materials have been biobanked. Specific authorisation in the informed consent is mandatory.		
**Disease Specific Use**	**Obligated**	**Whole of Resource**
Participants can choose if samples may be used ONLY for research projects on the participant’s disease or for other research projects on other diseases, too.		
**Geographical Area**	**Permitted**	**Whole of Resource**
The use, in countries not covered by GDPR, requires specific authorisation included in the informed consent.		
**Profit Motivated Use**	**Permitted**	**Part of Resource**
Depending on consent of the participant.		
**Regulatory Jurisdiction**	**Obligated**	**Whole of Resource**
Use of data is only allowed where the EU General Data Protection Regulation applies.		
**Research Use**	**Obligated**	**Whole of Resource**
Use for research purposes in the field of the disease for which the biological materials have been stored in the biobank. Most represented diseases are rare metabolic disorders, chromosome disorders, neurological diseases and overgrowth disorders.		
**Use As Control**	**Obligated**	**Whole of Resource**
Where the proposed use is relating to the field of the disease for which the biological materials have been biobanked.

**Table 4:** Process type CCE terms with further elaboration.

CCE Term	Rule	Scope
Further parameters		
**Collaboration**	**No Requirements**	**Part of Resource**
The collaboration is evaluated when appropriate		
**Ethics Approval**	**Obligated**	**Whole of Resource**
The requester must provide the following: protocol number, date and name of ethical committee/review board.		
**Fees**	**Obligated**	**Whole of Resource**
Fees apply when used for commercial or for-profit purposes.		
**Publication**	**Obligated**	**Whole of Resource**
Access to the samples is only for research purposes, measurable through the publication of research results. Each biobank that is used to perform a study must be mentioned in the Methods section of said publication.		
**Return Of Incidental Findings**	**Permitted**	**Whole of Resource**
The health and wellness of the participant (and his/her family) shall be safeguarded.		
**Return Of Results**	**Obligated**	**Whole of Resource**
Required for uses that overlap with the interests of the supplying institution.		
**(Re-)Identification Of Individuals Mediated By The Resource Provider**	**Obligated**	**Whole of Resource**
Informed consent should facilitate a continuous dialogue with participants to inform them about their disease. The way of re-identification should be compliant with the GDPR UE 2016/679 and with the participant’s choices.		
**(Re-)Identification Of Individuals Without The Involvement Of The Resource Provider**	**Permitted**	**Whole of Resource**
Informed consent should facilitate a continuous dialogue with participants and inform them about the disease. The way of re-identification should be compliant with GDPR UE 2016/679 and with the participant’s choices.		
**Time Period**	**Obligated**	**Whole of Resource**
The recipient is permitted to use the data for no more than 1 year after the agreed completion date; to permit the preparation of the results for publication.		
**User Authentication**	**Obligated**	**Whole of Resource**
To request biological materials, the recipient must register on the resource website by completing the specified form.		

## Discussion

In this work we have developed, validated, and recommend the Common Conditions of use Elements (CCEs): a set of conditions of use concepts that are atomic, non-directional, and which should be widely useful as a foundational layer in support of many aspects of data and sample sharing. The CCE list was devised by extensive discussion and alpha testing with many community members, followed by real-world testing in terms of creating general and detailed Policy Profiles. This latter work employed online software to utilise CCEs in the context of the DUC Schema for structuring information governance metadata.

We took a different approach to previous initiatives. Consent Codes aimed to provide a set of controlled terms that could be used to represent participant consent for uses in specific types of research. DUO expanded this to include general use conditions but focused on data only. Further the designers of DUO constructed it as a series of interconnected terms that formed a formal ontology. This means that care must be taken to ensure that a desirable term is present in the correct context within the DUO ontology.

ADA-M took an approach wherein each term was specific and separate. The ADA-M terms were split into two main categories: those that related to “Permissions” for specific uses and those that related to “Terms” of use. Like the current work these were paired with a rule base that either permitted or forbid the specific permission term or stating whether a given Term was true or not. CCEs (when used within a rule base such as the Digital Use Conditions DUC Schema) seek to simplify the approach taken by ADA-M. The idea of the two classes of terms is maintained but the rule base is simplified, and an optional free text field is added to allow elaboration of the full statement (CCE term, rule and scope) as required. While the free text is not currently able to be used as a filter, it does provide context to the end user when selected profiles are read.

The directional neutrality of CCEs ensures that uses covered by individual CCE terms could be permitted as well as forbidden using the appropriate rules when used in a data structure such as the DUC schema. While ADA-M had previously tried to take a similar approach, its’ complexity made it difficult to use, hampering its adoption. CCEs aimed to avoid this complexity by using simple independent atomic terms. The terms were also explained in plain English, with the aim of increasing their adoption by making them more accessible than some of the alternative approaches taken in this area.

Clearly, a series of such CCEs based on these principles would not, in and of themselves, be sufficient to describe a complete sharing or access policy. But that is not the objective of CCEs. Instead, the aim is to establish a widely relevant standardised set of atomic, non-directional, generic conditions of use concepts. These can then be employed as the basis for designing forms, contracts, tools and infrastructures that would be more innately inter-operable, and one would only need to elaborate the CCEs with directionality and other specifics to produce data sharing policies etc., that would all be structurally very similar and hence compatible and comparable.

Work is now underway to use the CCE model within the EJP-RD project, in particular to use them to gather Policy Profiles (that provide a summary of the use conditions, in a similar fashion to how the nutritional information for food is displayed in Europe) for many registries, biobanks and other online resources, and use these metadata to underpin data and sample discovery services and sharing activities. Additionally, other international initiatives have begun exploring ways to employ the CCE concept, not least BBMRI-ERIC^6^, GA4GH, the IMI EPND project^7^, and the FAIR community^8^. Some of this work entails extending the list of CCEs to suit specialised use cases, such as dynamic consent and support for GDPR considerations^9,10^. In the case of the FAIR community, CCE concepts are being tested for compatibility with semantic web models, such as Open Digital Rights Language (ODRL)^11^, to afford increased machine-readability. We anticipate versioning CCEs in a public manner, as these real-world activities progress. As this occurs, increasingly validated CCE concepts will ideally become included in formal ontologies, such as DUO or the Informed Consent Ontology^12^ (ICO). Table 5 illustrates the current overlap between CCEs (version 1.0) and DUO, in order to highlight gaps that could usefully be filled. All CCE terms are shown, along with their direct or indirect mapping to DUO.

**Table 5:** CCE terms and matching terms in DUO.

CCE Term	CCE mapping to DUO	DUO Term
Use As Control	Maps directly	Research Control
Clinical Research Use	Maps directly	Biomedical Research
Disease Specific Use	Maps directly	Disease Category Research
Geographical Area	To map, must add: ‘Rule = Permitted’	Geographical restriction
Research Use	To map, must add: ‘Rule = Permitted’	General research
Clinical Care Use	To map, must add: ‘Rule = Permitted’	Clinical Care Use
Return Of Results	To map, must add: ‘Rule = Obligated’	Return to database or resource
Collaboration	To map, must add: ‘Rule = Obligated’	Collaboration required
Time Period	To map, must add: ‘Rule = Obligated’	Time limit on use
Publication Moratorium	To map, must add: ‘Rule = Obligated’	Publication moratorium
Publication	To map, must add: ‘Rule = Obligated’	Publication required
User Authentication	To map, must add: ‘Rule = Obligated’	User specific restriction
Ethics Approval	To map, must add: ‘Rule = Obligated’	Ethics approval required
Commercial Entity	To map, must combine: Commercial Entity with ‘Rule = Permitted’ + Profit Motivated Use with ‘Rule = Forbidden’	Non-commercial use only
Profit Motivated Use	Commingled with Commercial Entity in DUO. (see above)	
Fees	Does not map to DUO	
Regulatory Jurisdiction	Does not map to DUO	
Return Of Incidental Findings	Does not map to DUO	
(Re-)Identification Of Individuals Without Involvement Of The Resource Provider	Does not map to DUO	
(Re-)Identification Of Individuals Mediated By The Resource Provider	Does not map to DUO	

Table 5 illustrates that whilst some DUO terms reflect composite data use conditions that bring together multiple related conditions of use as one ontology term, CCE would express similar conditions through the combination of multiple separate atomic terms. For example, in DUO, use by a commercial entity is commingled with for-profit use; jurisdiction and geographical location are likewise linked together. Further, select DUO terms are explicitly directional, and are intended to indicate that a certain behaviour is obligated in the use of data. Examples thereof include “collaboration required” and “time limit on use.” Conversely, CCEs would express these terms without implying directionality, which would enable users thereof to indicate the presence, absence, or explicit preclusion of each condition. The use of a lexicon composed of atomic and non-directional terms can enable communities to express the full range of permissions in their data using one common system. This can be leveraged to help communities with case-specific data governance needs to develop and tailor bespoke systems that are suitable to their needs, building upon a wider library of CCE terms. It also could enable organisations to use CCEs to enable interoperability across distinct, context-specific ontologies, allowing researchers to leverage context-specific ontologies of their choosing, whilst still enabling for the interoperable comparison of data governance conditions that have been expressed using multiple distinct ontologies, absent prior coordination.

## Methods

The system needed to be suitable for use by all the project partners involved, including those from research and healthcare sectors, as well as non-profit organisations. Given the broad range of activities undertaken by the various partners, the resulting use conditions needed to consider use cases outside of the traditional research community in which other systems had been developed.

### Defining CCE terms

CCE development work was undertaken within the project team and in conjunction with members of the rare disease community, to produce the version 1.0 model. This work involved many rounds of iterative testing and improvement of items in the CCE list and drafting and refining definitions for each. A reductionist approach was taken to devising the CCE terms.

This started with the capturing of concepts from various types of documents including Informed Consent Forms (ICF); data access policies (DAP); data/material transfer agreements (DTA/MTA) that were either accessible via the EJP-RD project (e.g., ICFs from ERNs and Biobanking community),or publicly available consents, DTA, and MTA, namely the Manchester Tissue Bank Material Transfer agreement; the UKRI consent form; Genomic England Cancer Research Consent Form; Cancer UK generic systemic anti-cancer treatment consent form; the Cancer research UK Immunotherapy consent form; the UKRI generic consent template; the UK Data services consent; the BMA/Law society - Consent template and Genomics England opt out ADDITONAL FINDINGS Q7 as well as ICF from the ERN and Biobanking community. Concepts were extracted and assessed against the CCE criteria detailed in Results. When terms did not meet these criteria, they were initially reviewed to determine if they could be adapted to meet the criteria (i.e., by breaking down into simpler atomic concepts and or removing the directionality), or otherwise rejected. The list was also checked to ensure that it contained elements that were identified as being of importance to rare disease patients based on a survey by McCormack *et al*.^13^. These concepts are listed in the Supplementary Table 1 together with the CCE terms to which they map to or the reason why they were not considered for integration in the final set of CCE terms.

In this way, we identified and optimised many facets of the CCE design, not least: revealing and addressing aspects that were likely to create misunderstanding; highlighting elements that needed to be split further into truly atomic concepts; bringing forward suggestions of frequently-needed concepts that were missing from the evolving CCE list; and, guiding subjective decisions about what was sufficiently ‘common’ and hence useful to go into a version 1.0 CCE list, versus what concepts were too specialised or infrequently used.

Progress was facilitated by means of a shared Excel document, and the use and stepwise development of custom software that included help texts and instructional videos, combined with regular feedback discussions with testers.

### Using CCE terms to produce CCE statements using the DUC schema and alpha testers assessment of their utility

The final set of CCE terms were evaluated for their utility by employing them as “condition terms” in the DUC schema. This schema allows each CCE term to be converted to a “statement” by the addition of a suitable rule (from DUC’s set of Obligatory, Permitted, Forbidden or No Requirement). To complete a CCE statement a “scope” of the rule was assigned specifying whether the statement applied to the “whole of the resource” or “part of the resource”.

### Web based tool for constructing DUC profiles using CCE statements

An online tool was developed^14^ (https://ducejprd.le.ac.uk) that includes a web based “wizard” interface that enabled alpha testers to select CCEs, and then enter Rules and Scope values. Alpha testers used the tool to make their resource level Policy Profiles. The tool also includes sections to provide details of the resource to which the use conditions apply to. Users could add as many or as few CCE statements to a profile as they wish. CCE statements could reuse or omit CCE terms as needed. CCE statements are independent of each other, and so the tool does not enable users to enter inter-statement dependencies. Profiles were reviewed, and their meaning was clarified with the alpha testers where required.

### Supplementary information

Supplementary Tables

## Data Availability

The original datasets were provided in confidence and to respect that confidentiality, we show the generated profiles as aggregated data in the manuscript (supplementary Table 2).

## Code availability

The code for the web-based tool for construction of a DUC/CCE profile (https://ducejprd.le.ac.uk) can be found at this public repository https://github.com/Cafe-Variome/DucCCE with read only access.
