# Supplementary material for: Common conditions of use elements. Atomic concepts for consistent and effective information governance
Source: Sci Data. 2024 May 8;11:465. doi: 10.1038/s41597-024-03279-z (PMC11078919; doi:10.1038/s41597-024-03279-z)
Supplement: Supplementary file 1 — Supplementary Tables [file 41597_2024_3279_MOESM1_ESM.pdf]

Table of Contents

Supplementary Table 1: Original use condition concepts …….Page 1

Supplementary Table 2: Feedback policy profiles……Page 4

Source of term	Core concept to be captured	Question (criterion/ process)	Mapped to final CCE term or reason for rejecting	CCE category that could be used for more granular terms with free text clarifications.
Manchester Brain Bank Form Section 3 - consent 1	Use as control material	why	Use as control	
UKRI Consent Form Question 3	Regulatory authorities' oversight to access asset	where	Regulatory Jurisdiction	
UKRI Consent Form Question 6	Use of held information to recontact participant	how	(Re-)Identification Of Individuals Mediated By The Resource Provider	
Manchester Brain Bank Form Section 3 Question 3	Use by pharmaceutical companies	who	not used (too granular)	Commercial Entity
Manchester Brain Bank Form Section 3 Question 3	Use by commercial companies	who	Commercial Entity	
Manchester Brain Bank Form Section 3 Question 4	Genetic analysis or study	why	Not used (too granular)	Research Use, Clinical Care Use, Clinical Research Use or Disease Specific Use
Manchester Brain Bank Form Section 3 question 5	Use outside of the UK	where	Geographical Area	
Manchester Brain Bank Form Section 3 question 6	Use for extended medical purposes - Reference material, medical education, audit and quality control	why	Clinical Research Use and Use As Control	
Manchester Brain Bank Form Section 4	Access to medical records, psychometric data and other medical trial data.	why	Not used (too granular)	None
UKRI Consent Form Question 3	Access by individuals from a named company	who	User Authentication	
UKRI Consent Form Question 3	Access by individuals from an NHS trust	who	User Authentication	
UKRI Consent Form Question 4	Sharing of anonymised data with other researchers.	what	Not used as it was thought most biobanks would not allow secondary sharing of their data. This was not contemplated for the purpose of discovery, , since most sharing needs an intermediate agreement in MTA, MTAs consulted forbid it	None
UKRI Consent Form Question 6	Used of participants data to provide information about health status.	how	(Re-)Identification Of Individuals Mediated By The Resource Provider	
ERN general consent form	Sharing of data amongst hospital based medical professionals within the EU	where	Geographical Area and Regulatory Jurisdiction plus explanatory free text.	
ERN general consent form	Sharing of data amongst hospital based medical professionals within the EU	where	Geographical Area and Regulatory Jurisdiction plus explanatory free text.	
ERN general consent form	Data sharing by consent of participant	how	Not used as it was thought most biobanks would not allow secondary sharing of their data. this was not contemplated for the purpose of discovery, , since most sharing needs an intermediate agreement in MTA, MTAs consulted forbid it	None
ERN general consent form	Recontacting the participant.	how	(Re-)Identification Of Individuals Mediated By The Resource Provider	
Manchester Brain Bank Form Section 3 - consent 2a	Research use	why	Research Use	
Manchester Brain Bank From Section 5B	Contact of nominated representative	how	(Re-)Identification Of Individuals Mediated By The Resource Provider (plus free tet for context)	
Doctoral course in Clinical Psychology consent to use recordings form	Use of session recordings for Supervision / Education purposes.	why	Not used (too granular)	Research Use , Clinical Care Use, Clinical Research Use or Disease Specific Use
IRAS consent template V3	Access to data collected by employees from a specified company	who	Commercial Entity and User Authentication	
BMA/ The law society consent form template.	Access by named legal representative for the data subject	who	User Authentication	
Determined as missing during coding work	Profit based use	why	Profit Motivated Use	
UKRI Consent Form Question 5	Informing the participant's GP of their involvement in study	why	Not used (too granular)	(Re-)Identification Of Individuals Mediated By The Resource Provider
Genomics England 100,000 Genomes consent form for adults with cancer	Inform the patients GP and other health care professional that they have joined the project.	why	Not used (too granular)	(Re-)Identification Of Individuals Mediated By The Resource Provider

Genomics England 100,000 Genomes consent form part 1 (consent 1) for adults with cancer	Recontact the participant to ask for more information related to the project	how	(Re-)Identification Of Individuals Mediated By The Resource Provider	
Genomics England 100,000 Genomes consent form part 1 (consent 1) for adults with cancer	Recontact the participant to ask for more samples	how	(Re-)Identification Of Individuals Mediated By The Resource Provider	
Genomics England 100,000 Genomes consent form part 1 (consent 1) for adults with cancer	Recontact the participant to Invite them to take part in other research	how	(Re-)Identification Of Individuals Mediated By The Resource Provider	
Genomics England 100,000 Genomes consent form part 1 (consent 1) for adults with cancer	Recontact the participate to provide updates about the project.	how	(Re-)Identification Of Individuals Mediated By The Resource Provider	
Genomics England 100,000 Genomes consent form part 2 for adults with cancer	Use of biosample for whole genome sequencing.	why	Not used (too granular)	Research Use, Clinical Care Use, Clinical Research Use or Disease Specific Use
Genomics England 100,000 Genomes consent form part 2 for adults with cancer	Use of biosample to determine gene expression.	why	Not used (too granular)	Research Use, Clinical Care Use, Clinical Research Use or Disease Specific Use
Genomics England 100,000 Genomes consent form part 2 for adults with cancer	Biosamples being sent outside of the country of in which the sample was taken for the purpose of specialist processing.	where	Geographical Area and Regulatory Jurisdiction plus explanatory free text.	
Genomics England 100,000 Genomes consent form part 2 (consent 1) for adults with cancer	Biosamples being sent outside of the country of in which the sample was taken for the purpose of specialist analysis.	where	Geographical Area and Regulatory Jurisdiction plus explanatory free text.	
Genomics England 100,000 Genomes consent form part 3 (consent 1) for adults with cancer	Project researcher access to full medical records, even those unrelated to the area under study (cancer in this case)		Not used (too granular)	(Re-)Identification Of Individuals Mediated By The Resource Provider, plus Research Use, Clinical Research Use or Disease Specific Use
Genomics England 100,000 Genomes consent form part 3 (consent 1) for adults with cancer (also in Rare Disease form)	Researcher use of patient's data to study conditions that doesn't affect the participant.		Not used (too granular)	Research Use, Clinical Research Use or Disease Specific Use
Genomics England 100,000 Genomes consent form part 3 (consent 1) for adults with cancer (also in Rare Disease form)	Continual collection of data from a participant's record even after death		Not used (too granular)	Various CCE terms depending on restrictions to specify the types of use then free text to explain the postmortem use.
Genomics England 100,000 Genomes consent form part 3 (consent 1) for adults with cancer (also in Rare Disease form)	Access by approved individuals (associated with the study or medical professionals) to the participant's information at any time	who	User Authentication	
Genomics England 100,000 Genomes consent form part 3 (consent 1) for adults with cancer	Data and samples being used by for-profit companies.	who/why	Commercial Entity and Profit Motivated Use	
Genomics England 100,000 Genomes consent form part 3 (consent 1) for adults with cancer (Also in Rare Disease form)	Removal or copying of participants data outside of the environment set up for the purpose of analysis.		Not used (too granular)	Various CCE terms depending on restrictions to specify the types of use then free text to explain the use outside of analysis environment.
Genomics England 100,000 Genomes consent form part 3 (consent 1) for adults with cancer	Running of tests on participants bio samples or data to identify the cause of their disease (cancer) - ***Core term diagnostic use***	why	Clinical care, (Re-)Identification Of Individuals Mediated By The Resource Provider, Return Of Results and/or Return Of Incidental Findings	
Genomics England 100,000 Genomes consent form part 3 (consent 1) for adults with cancer	Analysis of other information outside of the bio samples and data taken and any results obtained from them. - ***Core concept analysis of non-sample information and data.***	why	Not used (too granular)	Various CCE terms depending on restrictions to specify the types of use then free text to explain the data linkage aspect to existing data.
Genomics England 100,000 Genomes consent (for rare diseases) part 3	Access to other medical records outside of those held by the institution in question		Not used (too granular)	Various CCE terms depending on restrictions to specify the types of use then free text to explain the data linkage aspect to existing data.
Genomics England 100,000 Genomes consent (for rare diseases) part 3	Genetic analysis for diagnostic and care purposes.	why	Clinical Care Use plus free text to explain the genetic analysis component.	
Genomics England 100,000 Genomes consent (for rare diseases) part 4	Reporting of results to clinical care team	how	Return of Results	

Genomics England opt out 7B	Patient has requested NOT to receive additional findings	how	Return of incidental findings (forbidden)	
Genomics England opt out 7A	Patient has requested to receive additional findings	how	Return of incidental findings (obligated)	
vascern ICF	disease specific / research	why	Disease Specific Use and Research Use.	
vascern ICF	not for profit / research	why	Research Use and For profit use (Forbidden)	
vascern ICF	for profit / research	why	Research use and For profit use (Permitted)	
vascern ICF	Recontact to other reasons besides incidental findings	how	(Re-)Identification Of Individuals Mediated By The Resource Provider plus free text.	
vascern ICF	use for another research purpose (by the resource user)	why	Research Use (plus free text)	
vascern ICF	user access	who	User Authentication	
vascern ICF	Recontact to inform research results-incidental findings	how	(Re-)Identification Of Individuals Mediated By The Resource Provider, Return Of Results and/or Return Of Incidental Findings	
National Biobank of rare diseases - consent (informed section)	Restriction to specific uses/ purposes by patient (free text)	what	Use of appropriate CCE terms relating to use types with Forbidden rule and free text.	
National Biobank of rare diseases - consent (informed section)	Agreement to use by commercial companies/for-profit	who,why	Commercial Entity and Profit Motivated Use (Permtted)	
National Biobank of rare diseases - consent (informed section)	Induced pluripotent stem (iPS) cells studies		Not used (too granular)	Various CCE terms depending on restrictions to specify the types of use then free text to explain the data linkage aspect to existing data.
National Biobank of rare diseases - consent (informed section)	Recontact to inform research results-incidental findings	how	(Re-)Identification Of Individuals Mediated By The Resource Provider and Return Of Incidental Findings	
National Biobank of rare diseases - consent (informed section)	use for another research purpose (by the resource user)	why	Various CCEs to define permitted uses and free text to elaborate.	
National Biobank of rare diseases - consent (informed section)	Recontact to other reasons but incidental findings	how	(Re-)Identification Of Individuals Mediated By The Resource Provider and Return Of Incidental Findings	
Biobank Network Material Transfer Agreement	use for another research purpose (by the resource user)	why	Research use, disease specific research use	
Biobank Network Material Transfer Agreement	Custodianship-storage, management- of sample or data subject to specific regulatory compliance (several laws)	where	Regulatory Jurisdiction	
Biobank Network Material Transfer Agreement	Data/Sample sharing to third party is forbidden		Not used as it was thought most biobanks would not allow secondary sharing of their data. this was not contemplated for the purpose of discovery, , since most sharing needs an intermediate agreement in MTA, MTAs consulted forbid it	Various CCE terms depending on restrictions to specify the types of use then free text to explain the data linkage aspect to existing data.
Biobank Network Material Transfer Agreement	Use of individual names provider/recipient forbidden except with written consent	what	Not used (composite process, directional)	(Re-)Identification Of Individuals Mediated By The Resource Provider and Return Of Incidental Findings and free text
Biobank Network Material Transfer Agreement	Reference to provider in publications	what	Collaboration	
Biobank Network Material Transfer Agreement	Period of confidentiality from the completion or termination of the Research	when	Time period	
Biobank Network Material Transfer Agreement	Re-identification is forbidden	how	(Re-)Identification Of Individuals Mediated/not mediated By The Resource Provider	
Biobank Network Material Transfer Agreement	Return or disposal of remaining samples upon termination	how	Not used (too broad)	
Biobank Network Material Transfer Agreement	Traceability is required		Not used (too broad)	
MTA	aliquot kept for future diagnostic purposes	what	Not used (too granular)	Collaboration and free text
MTA	aliquot of modified derivatives to provider	what	Not used (too granular)	Collaboration and free text
MTA	use of biological material on human subjects		Not used (too broad)	
MTA	pseudonymization		Not used (too broad)	
vascern Data Sharing Agreement	Shall not try to identify the subject of data.	how	(Re-)Identification Of Individuals Mediated By The Resource Provider and Return Of Incidental Findings	
vascern Data Sharing Agreement	RECIPIENT shall implement appropriate technical and organizational measures to meet the requirements for data controllers of the APPLICABLE DATA PROTECTION LAW	where, how	regulatory jurisdiction	
vascern Data Sharing Agreement	RECIPIENT shall appropriately acknowledge PROVIDER and PROVIDER'S SCIENTIST as contributor of the DATA	what	collaboration	
vacsern data sharing agreement	data use limitation		Not used (too broad)	

Supplementary Table 2: Feedback policy profiles

Profile submitter<br><br>CCE Term[a]	Biobank	Biobank	Biobank	Biobank	Registry	Registry	Registry	Data Platform (FDP)
**Commercial Entity**		Forbidden Whole	Forbidden Whole	Permitted Whole	Permitted Part	Permitted Part		
**Geographical Area**		Obligated Whole	Obligated Whole	Permitted Whole				
**Regulatory Jurisdiction**		Obligated Whole	Obligated Whole	Permitted Whole	Obligated Whole	Obligated Whole	Obligated Whole	
**Research Use**	Permitted Whole	Obligated Whole	Obligated Whole	Permitted Whole	Permitted Part			Permitted Whole
**Clinical Care Use**		Obligated Whole		Permitted Whole	Permitted Whole			
**Clinical Research Use**		Obligated Whole	Permitted Whole	Permitted Whole	Permitted Whole			
**Disease Specific Use**		Obligated Whole	Permitted Whole	Obligated Whole	Permitted Whole			
**Use As Control**		Permitted Whole	Permitted Whole	Permitted Whole	Permitted Whole			
**Profit Motivated Use**		Permitted Whole	Forbidden Whole	Forbidden Whole	Permitted Part	Permitted Part	Permitted Part	
**Time Period**		Obligated Whole	Obligated Whole	Obligated Whole	Obligated Whole			
**Collaboration**		Obligated Whole	Obligated Whole		Permitted Whole			
**Fees**		Obligated Whole	Obligated Whole	Obligated Whole	Obligated Whole			
**Return Of Results**		Obligated Whole	Obligated Whole	Obligated Whole	Obligated Whole			

**Return Of Incidental Findings**		Obligated Whole	Obligated Whole	Obligated Whole	Permitted Part			
**(Re-)Identification Of Individuals Without Involvement Of The Resource Provider**		Permitted Whole	Forbidden Whole	Forbidden Whole	Forbidden Whole	Forbidden Whole	Forbidden Whole	
**(Re-)Identification Of Individuals Mediated By The Resource Provider**		Obligated Whole	Obligated Whole	Permitted Whole	Permitted Whole	Permitted Part		
**Publication Moratorium**		Obligated Whole	Obligated Whole					
**Publication**		Obligated Whole	Obligated Whole	Obligated Whole				
**User Authentication**		Obligated Whole	Obligated Whole	Obligated Whole				
**Ethics Approval**		Obligated Whole	Obligated Whole	Obligated Whole				

Directionality options: “Forbidden”, “Obligated”, “Permitted”.
Grey cells: CCE not used in that Policy Profile
Scope options: “Whole” (CCE + directionality applies to the whole of the resource), “Part” (CCE + directionality applies to part of the resource).
The profiles show different approaches adopted by the resources. Some wanted to be rather comprehensive, whereas others just wanted to state non-allowed forms of use.
